# Unmasking May-Thurner Syndrome: A Case Report of Massive Deep Vein Thrombosis

**DOI:** 10.7759/cureus.56979

**Published:** 2024-03-26

**Authors:** Khalid A Alshehri, Adnan A Bahashwan, Abdulaziz Y Fakieha, Hatem E Alkhamisi, Mohammed M Albladi

**Affiliations:** 1 General Surgery, King Faisal Specialist Hospital and Research Centre, Jeddah, SAU; 2 General Surgery, International Medical Center, Jeddah, SAU; 3 General Surgery, King Faisal Hospital, Taif, SAU; 4 General Practice, King Abdulaziz Medical City, Jeddah, SAU

**Keywords:** anatomical variations, anticoagulation therapy, catheter-directed thrombolysis, iliac vein compression, pulmonary embolism, may-thurner syndrome, deep venous thrombosis

## Abstract

Deep venous thrombosis is a significant medical condition that results in life-threatening complications such as pulmonary embolism. Various factors can contribute to the formation of deep venous thrombosis, including prolonged immobility, surgery, and specific health conditions. May-Thurner syndrome is an underrecognized cause of deep venous thrombosis due to the compression of the left common iliac vein by the right common iliac artery. It poses diagnostic challenges due to its varied clinical presentations. This report discusses a 42-year-old female with no notable medical history who presented with acute onset of left leg swelling, pain, and discoloration. Despite the absence of common risk factors for deep venous thrombosis, investigations revealed a massive left-sided deep venous thrombosis. Additional imaging studies revealed the diagnosis of May-Thurner syndrome, manifesting as significant compression of the left common iliac vein. The patient underwent anticoagulation therapy, catheter-directed thrombolysis, and stent placement, resulting in symptomatic improvement and no recurrence over a six-month follow-up period. This case underscores the necessity of considering anatomical variations like May-Thurner syndrome in patients with unexplained deep venous thrombosis, particularly without typical risk factors. It highlights the importance of a comprehensive diagnostic approach, including advanced imaging techniques, to uncover underlying causes of deep venous thrombosis.

## Introduction

Deep vein thrombosis is a critical medical condition that can lead to life-threatening complications, such as pulmonary embolism, highlighting the importance of timely diagnosis and management [1]. The etiology of deep vein thrombosis is multifactorial, with risk factors including prolonged immobilization, surgery, malignancy, inherited thrombophilia, and hormonal therapy [2]. Despite its relatively common occurrence, certain cases of deep vein thrombosis arise from less common anatomical abnormalities, presenting a diagnostic challenge [1,2].

May-Thurner syndrome, also known as iliac vein compression syndrome, is one such underrecognized condition that predisposes individuals, especially young women, to left-sided deep vein thrombosis. This syndrome results from compression of the left common iliac vein by the overlying right common iliac artery, leading to chronic venous stasis and increased risk of thrombosis [3]. The clinical presentation of May-Thurner syndrome can vary widely, from asymptomatic to severe limb swelling and pain [3]. Given its nonspecific presentation and the potential for significant morbidity, awareness, and understanding of May-Thurner syndrome are crucial for healthcare providers [1,3]. This report describes a case of massive left-sided deep vein thrombosis where subsequent investigations revealed the underlying diagnosis of May-Thurner syndrome, highlighting the importance of considering anatomical variations in the differential diagnosis of unexplained deep vein thrombosis.

## Case presentation

We present a case of a 42-year-old female patient with no significant past medical history, who came to the emergency department complaining of a three-day history of progressive swelling, pain, and a bluish discoloration of her left leg. The onset of symptoms was acute, with the patient noting that she awoke with mild discomfort that worsened significantly over the following days. She denied any recent travel, surgery, trauma, or history of thromboembolic events. The patient also reported no use of tobacco, alcohol, or illicit drugs and had no family history of clotting disorders.

**Figure 1:**
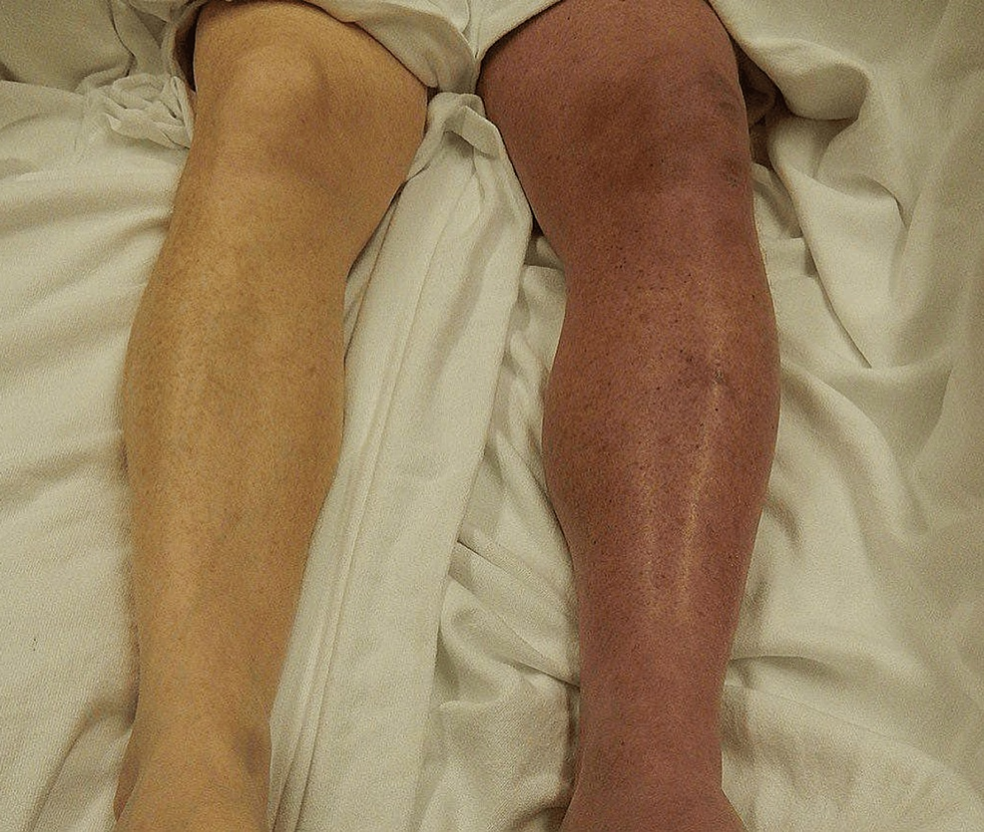
Comparative photograph of the patient's legs This photograph presents a side-by-side comparison of the patient's legs, highlighting the left leg's pronounced swelling, discoloration, and apparent venous distension relative to the right leg. The left leg demonstrates significant clinical manifestations of deep vein thrombosis, including increased circumference, a bluish-purple skin discoloration indicative of cyanosis, and visible venous congestion.

Upon physical examination, her left leg was notably swollen, measuring 3 centimeters larger in circumference than the right leg at the level of the thigh. Pitting edema extended up to the mid-thigh, and the skin over the left leg appeared cyanotic and was warm to the touch (Figure 1). The patient’s vital signs were within normal limits, with a heart rate of 78 beats per minute, blood pressure of 125/80 mmHg, respiratory rate of 16 breaths per minute, and a temperature of 98.6℉. The remainder of the physical examination was unremarkable.

Initial laboratory work-up showed a complete blood count with a hemoglobin level of 13.2 g/dL, a white blood cell count of 7.4 x 109/L, and a platelet count of 250 x 109/L. The coagulation profile revealed a prothrombin time of 13 seconds (international normalized ratio: 1.0) and an activated partial thromboplastin time of 30 seconds. The chemistry panel indicated normal kidney and liver function, with a creatinine level of 0.9 mg/dL and alanine aminotransferase of 22 U/L (Table 1).

**Table 1:** Laboratory findings on admission The results indicate a normal coagulation profile and no evidence of infection or significant renal or hepatic dysfunction, which is crucial in ruling out other causes of the patient's symptoms and focusing the diagnosis toward a thrombotic event.

Laboratory Test	Units	Result	Reference Range
Hemoglobin	g/dL	13.2	12.0 - 15.5
White Blood Cell Count	x 10^9^/L	7.4	4.0 - 11.0
Platelet Count	x 10^9^/L	250	150 - 400
Prothrombin Time	seconds	13	11 - 14
International Normalized Ratio	ratio	1.0	0.8 - 1.2
Activated Partial Thromboplastin Time	seconds	30	25 - 35
Creatinine	mg/dL	0.9	0.6 - 1.2
Alanine Aminotransferase	U/L	22	7 - 56
Aspartate Aminotransferase	U/L	24	8 - 48
Total Bilirubin	mg/dL	0.9	0.1 - 1.2
Albumin	g/dL	4.2	3.5 - 5.0
Blood Urea Nitrogen	mg/dL	14	6 - 20
D-dimer	mg/L	0.8	0 - 0.5

**Figure 2:**
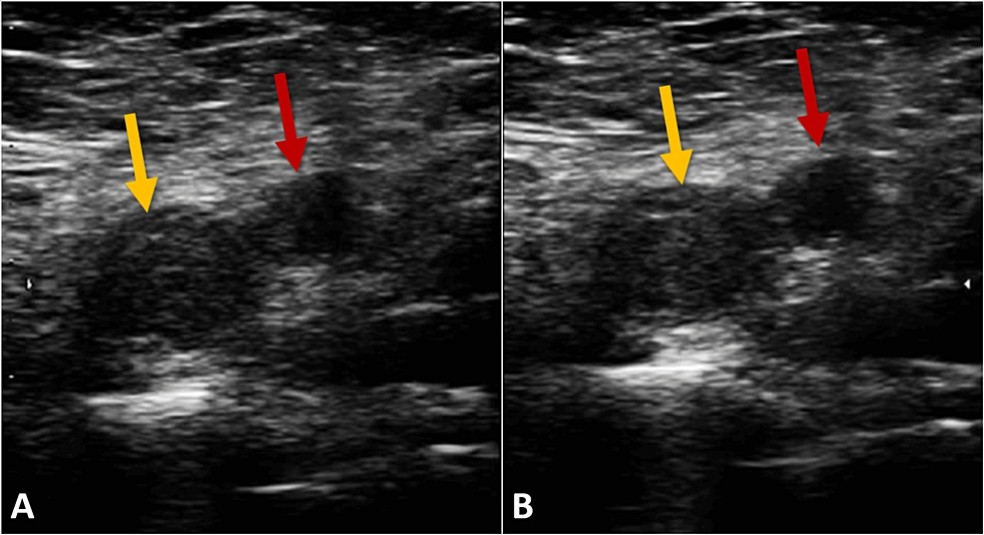
Color Doppler ultrasound of the femoral vein This color Doppler ultrasound image showcases the femoral vein (yellow arrow) and the femoral artery (red arrow), highlighting their normal anatomical positions without compression (A). The inability of the femoral vein to collapse under applied compression (B) indicates a critical diagnostic sign of deep vein thrombosis.

Suspecting deep vein thrombosis, a Doppler ultrasound of the lower extremities was performed, revealing an extensive thrombus in the left common and external iliac veins, which was indicative of a left-sided massive deep vein thrombosis (Figure 2). The unusual extent and location of the thrombosis, especially in a patient lacking overt risk factors, prompted further investigation into an underlying etiology.

**Figure 3:**
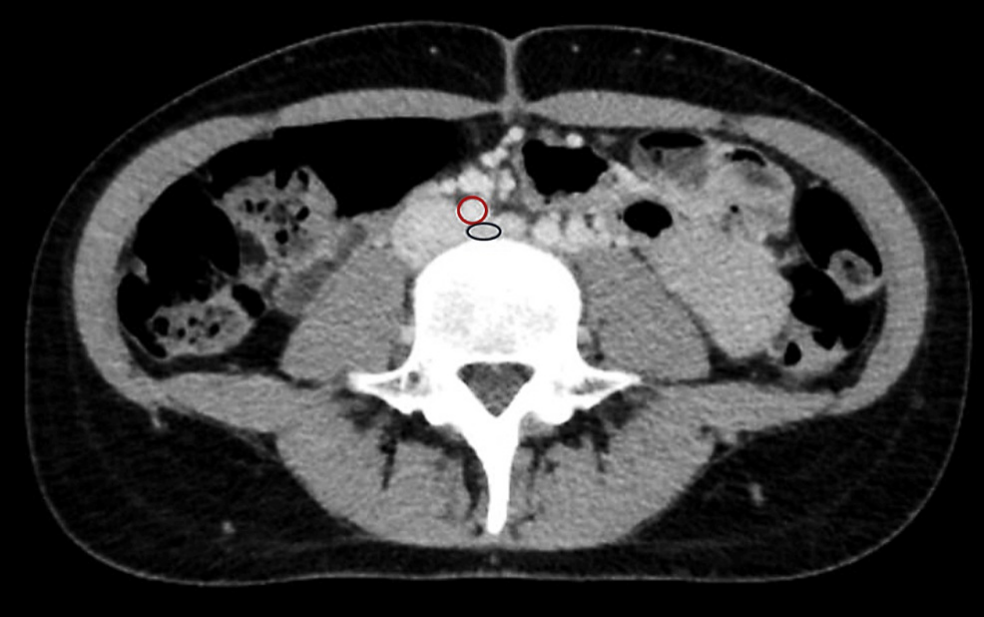
Computed tomography venogram showing May-Thurner syndrome A computed tomography venogram clearly highlights the significant compression of the left common iliac vein (blue circle) by the overlying right common iliac artery (red circle), providing a vivid visualization of the anatomical anomaly characteristic of May-Thurner syndrome. This illustrates the cause of the patient's deep vein thrombosis.

A computed tomography venogram was subsequently performed, which showed significant compression of the left common iliac vein by the overlying right common iliac artery, along with an extensive thrombus extending from the left common iliac vein into the inferior vena cava (Figure 3). These findings were diagnostic of May-Thurner syndrome, an anatomical condition where the left common iliac vein is compressed by the right common iliac artery, leading to venous stasis and thrombosis.

Management commenced with the initiation of anticoagulation therapy using low molecular weight heparin, with a transition to oral anticoagulants planned. Considering the severity of the thrombosis and the anatomical challenge posed by May-Thurner syndrome, the patient underwent catheter-directed thrombolysis followed by stent placement in the left common iliac vein to relieve the venous compression and prevent recurrent thrombosis. The hospital course was marked by a minor complication of mild bleeding at the catheter insertion site, which was managed conservatively.

The patient demonstrated significant symptomatic improvement, with a reduction in limb swelling. Over a six-month follow-up period, she remained symptom-free, with no evidence of recurrent thrombosis or post-thrombotic syndrome, as monitored through regular anticoagulation checks, Doppler ultrasound examinations, and clinical evaluations.

## Discussion

The complexities involved in diagnosing and managing May-Thurner syndrome, particularly in the context of a massive left-sided deep vein thrombosis, underscore a significant interplay between rare anatomical anomalies and prevalent vascular disorders. This case adds to the growing corpus of evidence emphasizing the need for a high degree of clinical suspicion for May-Thurner syndrome in patients presenting with unexplained lower extremity deep vein thrombosis, especially in the absence of traditional predisposing factors [2,4].

May-Thurner syndrome is characterized by the extrinsic compression of the left common iliac vein by the overlying right common iliac artery, predisposing individuals to venous stasis and thrombosis [1-3]. This phenomenon, initially described by May and Thurner in 1957, suggests that the chronic pulsation of the artery against the vein, coupled with compression against the lumbar vertebrae, leads to intimal hyperplasia and subsequent venous obstruction [3,4].

The differential diagnosis for deep vein thrombosis includes a variety of conditions such as cellulitis, lymphedema, and ruptured Baker's cyst [2-5]. However, in cases of left-sided deep vein thrombosis lacking apparent risk factors, the differential diagnosis narrows, emphasizing the relevance of May-Thurner syndrome. The clinical similarities between these conditions and May-Thurner syndrome-associated deep vein thrombosis often obscure the diagnostic pathway, underscoring the necessity for high clinical suspicion and the appropriate use of diagnostic imaging [4,5].

The diagnostic process in this case highlights the essential role of imaging in revealing the underlying cause of deep vein thrombosis. While Doppler ultrasound remains the first-line diagnostic tool for deep vein thrombosis, its limitations in visualizing the pelvic veins necessitate further imaging to identify May-Thurner syndrome [2-4]. Here, computed tomography venography was instrumental in confirming the presence of deep vein thrombosis and in identifying the anatomical compression indicative of May-Thurner syndrome.

Managing deep vein thrombosis induced by May-Thurner syndrome requires a nuanced approach that addresses both the immediate thrombotic event and the underlying venous compression [1,4]. This case illustrates an integrated management strategy encompassing anticoagulation, catheter-directed thrombolysis, and venous stenting. Literature suggests that while anticoagulation is crucial for preventing further thrombus formation, it does little to correct the underlying venous obstruction [2-4]. Catheter-directed thrombolysis and stenting not only address acute symptoms but also aim to prevent long-term complications by restoring venous flow [3,5].

## Conclusions

In conclusion, this case not only sheds light on the diagnostic and therapeutic challenges associated with May-Thurner syndrome but also emphasizes the critical need for awareness of anatomical considerations in vascular medicine. By detailing the relationship between anatomical anomalies and thrombotic risk, this discussion contributes to a deeper understanding of May-Thurner syndrome and advocates for a more comprehensive and aggressive management strategy. Future research should aim to refine diagnostic criteria, improve management guidelines, and clarify the long-term outlook for patients with May-Thurner syndrome, thereby enhancing patient outcomes in this underrecognized population.
